# Cytotoxicity of Different Concentrations of Three Root Canal Sealers on Human Mesenchymal Stem Cells

**DOI:** 10.3390/biom8030068

**Published:** 2018-08-01

**Authors:** Sara A. Alsubait, Reem Al Ajlan, Hala Mitwalli, Nour Aburaisi, Amer Mahmood, Manikandan Muthurangan, Randa Almadhri, Musaad Alfayez, Sukumaran Anil

**Affiliations:** 1 Department of Restorative Dental Science, College of Dentistry, King Saud University, Riyadh 11611, Saudi Arabia; 2 College of Dentistry, King Saud University, P.O. Box 85676, Riyadh 11611, Saudi Arabia; reem-alajlan@hotmail.com (R.A.A.); hala.mitwalli@gmail.com (H.M.); noursamer92@gmail.com (N.A.); 3 Stem Cell Unit, Department of Anatomy, College of Medicine, King Saud University, Riyadh 11461, Saudi Arabia; ammahmood@ksu.edu.sa (A.M.); coralmani@gmail.com (M.M.); r.i.almadhri@gmail.com (R.A.); alfayez@ksu.edu.sa (M.A.); 4 Department of Periodontics, Saveetha Dental College and Hospitals, Saveetha University, Poonamallee High Road, Chennai 600077, India; drsanil@gmail.com

**Keywords:** AH Plus, BioRoot RCS, cytotoxicity, Endosequence BC, mesenchymal stem cells

## Abstract

This study assessed the dose-dependent effect on the cytotoxicity of BioRoot RCS (BR) and Endosequence BC (BC) sealers in human bone marrow mesenchymal stem cells (hMSCs) compared to those of the AH Plus sealer. Cells were exposed to different dilutions of extracts from freshly prepared sealers (1:2, 1:8, 1:32). Unexposed cells acted as the negative control. Cytotoxicity was evaluated by an alamar blue assay. Cell morphology was analyzed by using scanning electron microscopy after exposure to the different sealers’ extracts. Statistical analysis was performed using a one-way analysis of variance and the Bonferroni post hoc test (*p* < 0.05). The cytotoxicities of BC and BR were less than that of AH Plus. In the presence of 1:2 BR, the cell proliferation was significantly lower than the control. At 1:8 and 1:32 concentrations, both the tricalcium silicate sealers led to similar cellular proliferation. Cells in BC and BR sealers’ extracts spread better than those in AH Plus extract.

## 1. Introduction

The final stage of endodontic treatment is to obturate the canal space. Filling the root canal is classically performed using gutta-percha in combination with a root canal sealer [1]. Based on the main chemical composition, endodontic sealers can be classified into glass ionomer, zinc oxide–eugenol, resin, calcium hydroxide, silicone, and bioceramic-based root canal sealers. Bioceramic-based root canal sealers have been introduced to the market after the popularity of bioceramic cements, which are biocompatible [2] and bioactive [3,4]. Endosequence BC sealer (Brasseler USA, Savannah, GA, USA) is a premixed bioceramic sealer. According to the manufacturer, it is composed mainly of tri- and di-calcium silicates, zirconium oxide, and calcium phosphate. It requires the presence of water to set and harden. BC sealer exhibits biocompatibility and antibacterial activity [5,6].

Another bioceramic-based sealer, BioRoot RCS (Septodont, Saint Maur Des Fosses, France), is a hand-mixed sealer that is supplied as a powder and liquid. According to the manufacturer, the powder is composed mainly of tricalcium silicate and zirconium oxide. The liquid contains calcium chloride. BioRoot RCS showed a low cytotoxicity in human periodontal ligament cells and induced the secretion of osteogenic growth factors [7,8].

Clinically, sealers are introduced into canals in a fresh, unset state. They are designed to be confined within the canal; however, they might be extruded through the apical constriction or lateral canals [9]. Even without extrusion, sealers may release some chemical components to the periapical tissues [10]. To the best of our knowledge, few reports have evaluated the biological responses to Endosequence BC sealer [5,11], with none being conducted on freshly prepared BioRoot RCS. Furthermore, no studies directly compared the cytotoxicity between these two types of tricalcium silicate-containing root canal sealers. Therefore, the purpose of the current study was to assess the dose-dependent effect on the cytotoxicities of BioRoot RCS and Endosequence BC sealers on human bone marrow mesenchymal stem cells compared to that of the AH Plus sealer.

## 2. Materials and Methods

### 2.1. Sealers

BioRoot RCS, Endosequence BC sealer, and AH Plus Jet sealer (Dentsply DeTrey, Konstanz, Germany) were used in the present study.

### 2.2. Preparation of the Extracts

Endodontic sealers were prepared according to the manufacturers’ instructions. AH Plus Jet was mixed using the automixing syringe. Endosequence BC sealer is a premixed ready-to-use sealer. BioRoot was prepared by mixing one spoonful of powder with five drops of BioRoot liquid using a spatula for 60 s. The sealers’ elutes were prepared according to International Organization for Standardization (ISO) 10993-5 [12] and as described previously [13]. Briefly, 0.3 mL of freshly prepared sealer was dispensed at the well bottom of a 24-well plate (Corning^®^ Costar^®^ cell culture plates, Sigma Aldrich, St. Louis, MO, USA). The surface of the material was smoothed, and immediately after, 2 mL culture medium “growth medium” was added to each well. The medium consisted of Dulbecco’s Modified Eagle medium (DMEM, Gibco BRL, Karlsruhe, Germany), supplemented with 2 mmol/L l-glutamine (Gibco, Germany), 10% fetal bovine serum (Gibco, Germany), 100 U/mL of penicillin, 100 μg/mL of streptomycin, and 1% of non-essential amino acids. The plates were incubated for 24 h at 37 °C with 5% CO_2_. Afterwards, the extracts were filtered (0.2-μm pore size), diluted (1:2, 1:8, and 1:32) [5], aliquoted, and frozen at −20 °C. All experimental procedures were performed under aseptic conditions under a class II laminar flow hood (LabGard ES 425 Biological Safety Cabinet, NuAire^®^, Plymouth, MN, USA).

### 2.3. Cell Culture

Immortalized human bone marrow-derived mesenchymal stem cells (hMSCs) were used for all experiments in the present study [14]. The cell lines were developed at the Stem cell unit at King Saud University. Cells were grown in a “growth medium”. Cell cultures were maintained in a humidified atmosphere of 5% CO_2_ at 37 °C.

### 2.4. Test Groups

Four groups were included as follows:

1. AH Plus sealer group (AH Plus): Cells grown in medium conditioned by AH Plus sealer.
2. Endosequence BC sealer group (BC): Cells grown in medium conditioned by Endosequence BC sealer.
3. BioRoot RCS group (BR): Cells grown in medium conditioned by BioRoot sealer.
4. Control group (C): Cells cultured in growth medium.

### 2.5. Cytotoxicity Assay

Sealers were tested for possible effects on cell proliferation and metabolic activity using Alamar Blue Assay (AlamarBlue; AbD Serotec, Kidlington, UK). The reagent contains an indicator dye, which fluoresces in response to cell growth. Briefly, cells were seeded in a 96-well plate (1 × 10^4^ cells/well) and incubated for 24 h to allow cell attachment. Culture media was then aspirated from each well and replaced with 50 μL of the three different dilutions of sealers’ extracts and incubated for 1, 3, or 7 days. At the end of the incubation period, 10% alamar blue reagent was added to each well. Plates were further incubated for 4 hr. The fluorescence of each well was measured at an excitation wavelength of 530 nm and an emission wavelength of 590 nm with a fluorescence reader (BioTek^®^, Winooski, VT, USA). The data were gathered using the Gen5 Data Analysis Software (BioTek^®^, Winooski, VT, USA). The experiment was performed in three wells per condition, and each one was performed in triplicate.

### 2.6. Cellular Morphology

Morphological changes were evaluated using a scanning electron microscope (SEM) after 24 h exposure to the different sealers’ extracts. Briefly, cells were seeded (1.5 × 10^6^ cells/well) in 13-mm-diameter glass cover slides placed at the bottom of a 24-well plate. After 24 h, 0.4 mL of each sealer extract was added to the glass cover slides in the 24-well plate, while the control group had growth medium added to it instead. At the end of the incubation period, solutions were aspirated, slides were washed using phosphate buffered saline (PBS), and cells were fixated by adding 1 mL of 2.5% glutaraldehyde. Following 2 h of fixation, the cells were washed in 1 mL PBS for 5 min and were then post-fixed in 1% osmium tetroxide for 1 h. Finally, dehydration of the cells was performed using an ascending exchange of ethanol solution concentrations, 30%, 50%, 70%, 95%, 100%, for 5 min each. Final drying of the specimens was completed using a critical point method dryer with CO_2_. The glass-cover slides containing the cell specimens were seated, secured on metal stubs, and then gold sputtered. Specimens were then observed and photographed using SEM (JSM-6360 LV, JEOL Corp., Peabody, MA, USA). Digital images were acquired at 1000× for each sample.

### 2.7. Statistical Analysis

Data from Alamar Blue Assay were expressed as the mean ± standard deviation. One-way analysis of variance was used in combination with the Bonferroni post hoc test for data evaluation. Values of *p* < 0.05 were considered significant. Statistical analysis was performed using SPSS statistical software (version 16; SPSS Inc., Chicago, IL, USA).

## 3. Results

### 3.1. Alamar Blue

**Figure 1:**
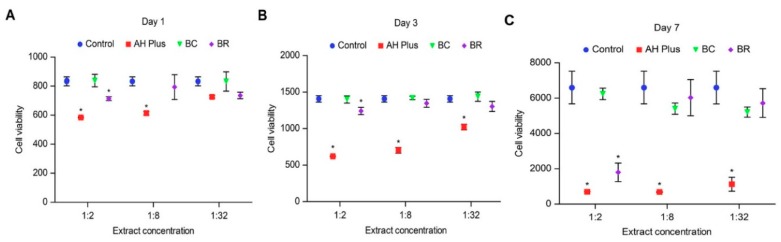
Cell viability of human bone marrow mesenchymal stem cells (hMSCs) cultures exposed to 1:2, 1:8, and 1:32 sealer extracts for (**A**) 1; (**B**) 3; and (**C**) 7 days. (BC—Endosequence BC, BR—BioRoot RCS) * A statistically significant difference compared with the control group (*p* < 0.05).

Figure 1 presents the cell viabilities of the hMSCs after treatment with each sealers’ extract on days 1, 3, and 7. At each time point, the number of cells in AH Plus was significantly lower than the control group (*p* < 0.05); however, in day 1, there was no significant difference in cell viability between 1:32 dilution of AH Plus and the control (*p* = 0.06). No significant difference was detected in cell viability between the BC sealer and the control at any time point. In the presence of 1:2 BR, the cell proliferation was significantly lower than the control at day 1 (*p* = 0.01), 3 (*p* = 0.03), and 7 (*p* = 0.03). No significant difference in cell viability were detected between 1:8, or 1:32 BR and the control after 1, 3, and 7 days of incubation.

For each concentration at each time point, the number of cells in AH Plus was significantly lower than the tricalcium silicate-based sealer groups; however, at day 1, cell proliferation in the 1:32 dilution of AH Plus was not significantly different from 1:32 BC (*p* = 0.06) or 1:32 BR (*p* = 1.00). Furthermore, at day 7, there was no significant difference in cell proliferation in the presence of 1:2 AH Plus or 1:2 BR (*p* = 0.32).

Comparing the tricalcium silicate-based sealers, at 1:2 dilution, cells incubated with BC showed significantly higher cell viabilities than 1:2 BR at day 1 (*p* = 0.01), 3 (*p* = 0.03), and 7 (*p* = 0.00). At 1:8 and 1:32 concentrations, both sealers led to similar cellular proliferations on days 1, 3, and 7.

### 3.2. Scanning Electron Microscope

**Figure 2:**
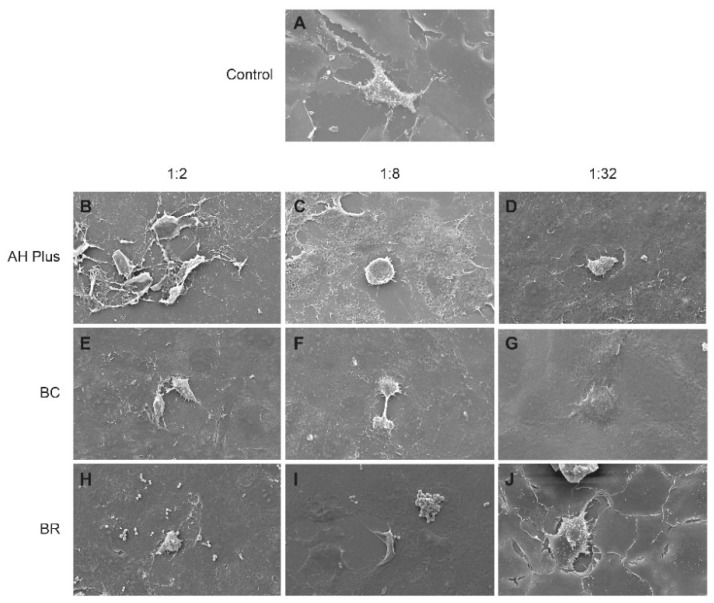
Scanning electron micrographs of the morphology of hMSCs control (**A**) exposed to (**B**,**E**,**H**) 1:2, (**C**,**F**,**I**) 1:8, and (**D**,**G**,**J**) 1:32 (**B**–**D**) AH Plus, (**E**–**G**) Endosequence BC, and (**H**–**J**) BioRoot sealer extracts for 24 h (×1000). Scale bars = 10 μm.

SEM examination after 24 h revealed different cell morphology in hMSCs when exposed to various sealers’ extracts (Figure 2). Cells in the control group appeared to be flat and amorphous in shape (Figure 2A). Cells in AH Plus specimens were detached at the 1:2 dilution level (Figure 2B). At 1:8 dilution, cells appeared rounded in shape with undefined edges, and some cytoplasmic extensions (Figure 2C). Cells were arranged more into sheets at 1:32 dilution level (Figure 2D). In contrast, hMSCs in BC sealer group were flat in appearance with irregular margins, indicating stronger cellular adhesion. The pattern of spreading appeared to increase with greater dilution levels (Figure 2E–G). Some cells in BR specimens were round in 1:2 and 1:8 dilution levels (Figure 2H,I). In 1:32 dilution, cellular spreading was similar to that in the control group (Figure 2J).

## 4. Discussion

This study was designed to evaluate the cytotoxicity of two bioceramic-based root canal sealers. AH Plus was included for comparison because it is widely used in endodontics and it is considered to be the gold standard against which all new sealers are compared [5,15]. Endodontic sealers might leak out some products to the periapical area. The concentrations of such elutes are progressively lowered because they are being cleared by the extracellular fluids [13,16]. Therefore, in the current study, different concentrations of extracts were prepared from freshly prepared sealers to provide information on the dose-dependent effects of the diffusible components on hMSCs. The in vitro tests are designed to evaluate the initial biological responses of biomaterials. Alamar blue assay has been used in dental research to evaluate the cell viability [17,18]. The advantages of alamar blue include its simplicity and the use of a non-toxic and non-radioactive compound [19]. In the present study, the freshly mixed AH Plus was cytotoxic in a concentration-dependent manner. This might be caused by the minimum release of formaldehyde from amines added to accelerate the epoxy polymerization [20].

Previous studies have demonstrated the biocompatibility of the BC sealer, after complete setting, with MG63 osteoblast-like cells [21] and human periodontal ligament cells [22]. Zhou et al. showed excellent biocompatibility of fresh BC sealer to human gingival fibroblasts [5]. Consistent with Zhou et al., the results of alamar blue showed a non-cytotoxic effect of BC sealer to hMSCs. However, these results are inconsistent with those of Loushine et al., in which BC Sealer was cytotoxic to mouse osteoblasts in the first five weeks [23]. These discrepant findings may be related to differences in experimental conditions, including the cell line that was used, as well as the manner in which extracts were presented to the cells.

The results of the present study showed that extracts from BR had dose-dependent effects on the proliferation of hMSCs. Collado-Gonzalez et al. reported that BR showed good biocompatibility with periodontal ligament cells [7]. Our results partially concur with the previous findings. At 1:8 and 1:32 concentrations, BR was biocompatible. At a higher concentrations, 1:2 BR was more cytotoxic to hMSCs compared to the control. Discrepancies could be related to the differences in methodology. Collado-Gonzalez et al. evaluated the effect of BR on human periodontal ligament cells after final setting. However, sealers may have the potential to release byproducts before complete setting. These byproducts might cause initial toxicity on the proliferation of hMSCs that gradually decreases with the dilution of these eluates. In addition, the composition of BR resembles that of Biodentine (Septodont, Saint Maur Des Fosses, France), a tricalcium silicate-based cement, which can be used as a dentine replacement material in the crown and in the root. A drop in cell viability after exposure to high concentrations of Biodentine extracts was previously reported [24,25]. It has been stated that this could be caused by the release of calcium ions and the increase in pH [26].

Cytotoxicity can be evaluated by characterization of cellular morphology under SEM. In our study, hMSCs exposed to different extracts of the BC and BR sealers were more flattened and confluent compared with hMSCs on AH Plus. These results suggest that these sealers promote greater attachment and are more biocompatible than AH Plus. Furthermore, high concentrations of BR had some round cells, indicating the toxic potential of the BR. These findings are consistent with the cell viability results. In general, the tricalcium silicate sealers showed higher cell viability than AH Plus. The composition of endodontic materials plays an important role in their biocompatibility. Endosequence BC and BioRoot RCS sealers contain tricalcium silicate, which are biocompatible [27].

On the basis of our findings, BC has good biocompatibility in all three different concentrations. BC is premixed tricalcium silicate-based sealer that eliminates the potential of heterogeneous consistency during on-site mixing. The in vitro methods used in the present study have the advantages of being simple and amenable to the control of experimental variables. A major disadvantage is that they do not take into account that the host defense mechanisms may affect clinical outcomes. The results from this study might provide information that can aid the clinician in the selection of the best material to use in clinical practice. Further in vivo studies are necessary to investigate the biocompatibility of these sealers.

## 5. Conclusions

Within the limitations of the present study, it may be concluded that BC and BR sealer extracts are less toxic to hMSCs compared with AH Plus. However, at higher concentrations, BR had less cytocompatibility than BC.

## References

[1] Ørstavik D. (2005). Materials used for root canal obturation: Technical, biological and clinical testing. Endod. Top..

[2] Keiser K., Johnson C., Tipton D. (2000). Cytotoxicity of mineral trioxide aggregate using human periodontal ligament fibroblasts. J. Endod..

[3] Sarkar N.K., Caicedo R., Ritwik P., Moiseyeva R., Kawashima I. (2005). Physicochemical basis of the biologic properties of mineral trioxide aggregate. J. Endod..

[4] Grazziotin-Soares R., Nekoofar M.H., Davies T., Hubler R., Meraji N., Dummer P.M.H. (2017). Crystalline phases involved in the hydration of calcium silicate-based cements: Semi-quantitative Rietveld X-ray diffraction analysis. Aust. Endod. J..

[5] Zhou H.M., Du T.F., Shen Y., Wang Z.J., Zheng Y.F., Haapasalo M. (2015). In vitro cytotoxicity of calcium silicate-containing endodontic sealers. J. Endod..

[6] Candeiro G.T., Moura-Netto C., D’Almeida-Couto R.S., Azambuja-Junior N., Marques M.M., Cai S., Gavini G. (2015). Cytotoxicity, genotoxicity and antibacterial effectiveness of a bioceramic endodontic sealer. Int. Endod. J..

[7] Collado-Gonzalez M., Garcia-Bernal D., Onate-Sanchez R.E., Ortolani-Seltenerich P.S., Lozano A., Forner L., Llena C., Rodriguez-Lozano F.J. (2017). Biocompatibility of three new calcium silicate-based endodontic sealers on human periodontal ligament stem cells. Int. Endod. J..

[8] Camps J., Jeanneau C., El Ayachi I., Laurent P., About I. (2015). Bioactivity of a calcium silicate-based endodontic cement (BioRoot RCS): Interactions with human periodontal ligament cells in vitro. J. Endod..

[9] Ricucci D., Langeland K. (1998). Apical limit of root canal instrumentation and obturation, part 2. A histological study. Int. Endod. J..

[10] Huang T.H., Ding S.J., Hsu T.Z., Lee Z.D., Kao C.T. (2004). Root canal sealers induce cytotoxicity and necrosis. J. Mater. Sci. Mater. Med..

[11] Zoufan K., Jiang J., Komabayashi T., Wang Y.H., Safavi K.E., Zhu Q. (2011). Cytotoxicity evaluation of Gutta flow and endo sequence BC sealers. Oral Surg. Oral Med. Oral Pathol. Oral Radiol. Endod..

[12] ISO (2009). Biological Evaluation of Medical Devices—Part 5: Tests for In Vitro Cytotoxicity.

[13] Rodrigues C., Costa-Rodrigues J., Capelas J.A., Fernandes M.H. (2013). Long-term dose- and time-dependent effects of endodontic sealers in human in vitro osteoclastogenesis. J. Endod..

[14] Elsafadi M., Manikandan M., Atteya M., Hashmi J.A., Iqbal Z., Aldahmash A., Alfayez M., Kassem M., Mahmood A. (2016). Characterization of cellular and molecular heterogeneity of bone marrow stromal cells. Stem Cells Int..

[15] Miletic I., Devcic N., Anic I., Borcic J., Karlovic Z., Osmak M. (2005). The cytotoxicity of RoekoSeal and AH plus compared during different setting periods. J. Endod..

[16] Barros J., Costa-Rodrigues J., Lopes M.A., Pina-Vaz I., Fernandes M.H. (2014). Response of human osteoblastic and osteoclastic cells to AH plus and pulp canal sealer containing quaternary ammonium polyethylenimine nanoparticles. J. Endod..

[17] Corral Nunez C.M., Bosomworth H.J., Field C., Whitworth J.M., Valentine R.A. (2014). Biodentine and mineral trioxide aggregate induce similar cellular responses in a fibroblast cell line. J. Endod..

[18] Alkahtani A., Alkahtany S.M., Mahmood A., Elsafadi M.A., Aldahmash A.M., Anil S. (2014). Cytotoxicity of QMix™ endodontic irrigating solution on human bone marrow mesenchymal stem cells. BMC Oral Health.

[19] Rampersad S.N. (2012). Multiple applications of alamar blue as an indicator of metabolic function and cellular health in cell viability bioassays. Sensors.

[20] Cohen B.I., Pagnillo M.K., Musikant B.L., Deutsch A.S. (1998). Formaldehyde evaluation from endodontic materials. Oral Health.

[21] Zhang W., Li Z., Peng B. (2010). Effects of iRoot SP on mineralization-related genes expression in MG63 cells. J. Endod..

[22] Chang S.W., Lee S.Y., Kang S.K., Kum K.Y., Kim E.C. (2014). In vitro biocompatibility, inflammatory response, and osteogenic potential of 4 root canal sealers: Sealapex, sankin apatite root sealer, MTA fillapex, and iRoot SP root canal sealer. J. Endod..

[23] Loushine B.A., Bryan T.E., Looney S.W., Gillen B.M., Loushine R.J., Weller R.N., Pashley D.H., Tay F.R. (2011). Setting properties and cytotoxicity evaluation of a premixed bioceramic root canal sealer. J. Endod..

[24] Gomes-Cornelio A.L., Rodrigues E.M., Salles L.P., Mestieri L.B., Faria G., Guerreiro-Tanomaru J.M., Tanomaru-Filho M. (2017). Bioactivity of MTA plus, biodentine and an experimental calcium silicate-based cement on human osteoblast-like cells. Int. Endod. J..

[25] Rodrigues E.M., Gomes-Cornelio A.L., Soares-Costa A., Salles L.P., Velayutham M., Rossa-Junior C., Guerreiro-Tanomaru J.M., Tanomaru-Filho M. (2017). An assessment of the overexpression of BMP-2 in transfected human osteoblast cells stimulated by mineral trioxide aggregate and biodentine. Int. Endod. J..

[26] Zanini M., Sautier J.M., Berdal A., Simon S. (2012). Biodentine induces immortalized murine pulp cell differentiation into odontoblast-like cells and stimulates biomineralization. J. Endod..

[27] Zhao W., Wang J., Zhai W., Wang Z., Chang J. (2005). The self-setting properties and in vitro bioactivity of tricalcium silicate. Biomaterials.

